# Small Bowel Dissemination of Coccidioidomycosis

**DOI:** 10.1155/2015/403671

**Published:** 2015-01-05

**Authors:** Shengmei Zhou, Yanling Ma, Parakrama Chandrasoma

**Affiliations:** ^1^Department of Pathology and Laboratory Medicine, Children's Hospital Los Angeles, MS 43, 4650 Sunset Boulevard, Los Angeles, CA 90027, USA; ^2^Keck School of Medicine, University of Southern California, Los Angeles, CA 90033, USA; ^3^Department of Pathology, LAC+USC Medical Center, Los Angeles, CA 90033, USA

## Abstract

Gastrointestinal coccidioidomycosis is extremely rare, with less than 10 cases reported in the literature. We report a case of small bowel dissemination of coccidioidomycosis in a 21-year-old African American male with a history of living in San Joaquin Valley. The patient presented with one week of abdominal pain, nausea, shortness of breath, intermittent fever, and sweat, and one month of abdominal distention. A chest radiograph revealed complete effusion of left lung. A computed tomography scan of the abdomen showed diffuse small bowel thickening and enhancement, as well as omental and peritoneal nodules, and ascites. The coccidioidal complement fixation titer was 1 : 256. The duodenal biopsy revealed many spherules filled with round fungal endospores. Later, blood fungal culture showed positivity for *Coccidioides immitis*. The final diagnosis is disseminated coccidioidomycosis involving lungs, blood, and duodenum. Despite aggressive antifungal therapy, the patient's clinical situation deteriorated and he succumbed to multisystem organ failure one and half months later. A high index of suspicion for gastrointestinal coccidioidomycosis should be maintained in patients from an endemic area presenting as abdominal distention and pain.

## 1. Introduction

Coccidioidomycosis is a growing problem in the endemic regions of Arizona and California [1]. Most cases of coccidioidal infection are completely asymptomatic, but approximately 30% present as a pulmonary process that may be difficult to distinguish from a bacterial community-acquired pneumonia [2]. This has led to underdiagnosis of coccidioidomycosis and inappropriate antibacterial therapy. Extrapulmonary disease develops in about 1 of 200 people infected with coccidioidomycosis and is associated with significant morbidity and mortality [3]. The common sites are the skin, meninges, bone and joints, and soft tissues [3]. The gastrointestinal tract has been uniquely spared from this fungal infection. To the best of our knowledge, less than 10 cases of gastrointestinal dissemination of coccidioidomycosis have been reported in the literature [4–6]. We describe a case of small bowel dissemination of coccidioidomycosis in a 21-year-old male.

## 2. Case Presentation

**Figure 1:**
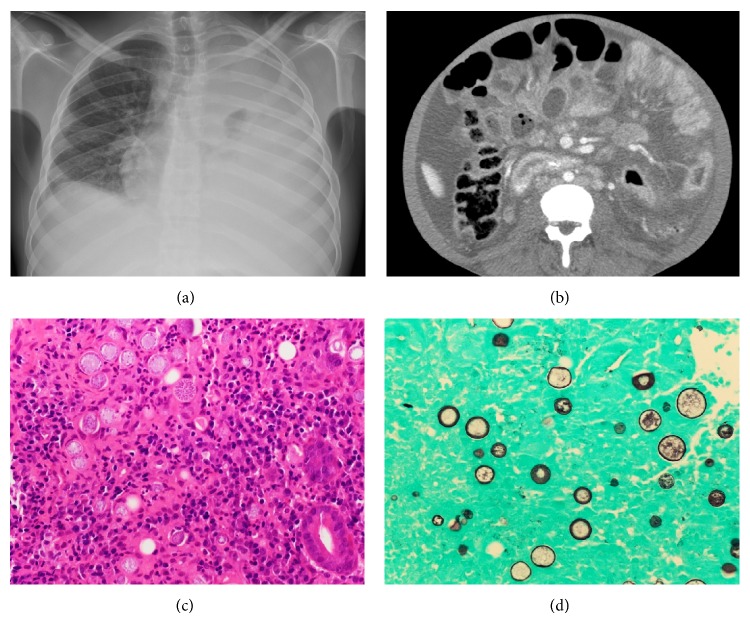
(a) A chest radiograph demonstrated complete opacity of the left lung and a small opacity in the right lower lobe. (b) CT scan showed diffuse small bowel thickening and enhancement. ((c) and (d)) Multiple spherules filled with round fungal endospores and scattered individual endospores were identified in the lamina propria of the duodenum ((c) H & E stain; (d) Gomori methenamine silver stain, original magnification ×400 for both).

A 21-year-old incarcerated (had been in jail for 7 months) African American male presented with one week of abdominal pain, nausea, shortness of breath, intermittent fever, and sweat, one month of abdominal distention, and a 30 lbs weight loss in the past year. Previous medical history included heavy alcohol abuse since the age of 10, asthma, and “arthritis” (pain in ankles and knees starting 9 months earlier). On physical exam, the patient was febrile up to 102.4 F. He appeared cachectic and had temporal wasting. Other significant physical exam findings included decreased breath sounds over the entire left lung and right lower lobe, abdominal distention with diffuse tenderness, and an enlarged supraclavicular lymph node. A chest radiograph revealed complete effusion of left lung (Figure 1(a)). Abdominal US showed enlarged fatty liver with ascites and an absent kidney, possibly because of nephrectomy and questionable retroperitoneal lymphadenopathy. An unsuccessful attempt was made to biopsy the enlarged supraclavicular lymph node. A CT of the abdomen showed diffuse small bowel thickening and enhancement (Figure 1(b)), as well as omental and peritoneal nodularity, and ascites. A duodenal biopsy was performed (hospital day 18).

An extensive work-up was done. The patient was found to have severe anemia (Hb 9.7 g/dL) and very low albumin (1.6 g/dL); malignancy such as multiple myeloma and lymphoma, autoimmune disease, and the following infections were ruled out: human immunodeficiency virus (HIV), hepatitis B and C, tuberculosis, histoplasmosis,* Cryptococcus*, and bacteria. Then, the coccidioidal complement fixation titer was found to be 1 : 256. The duodenal biopsy revealed many spherules filled with round fungal endospores throughout the wall, accompanied by chronic inflammation (Figure 1(c)). The spherules were stained positively with Gomori methenamine silver (Figure 1(d)). Later, blood fungal culture showed positivity for* Coccidioides immitis*. The final diagnosis is disseminated coccidioidomycosis involving lungs, blood, and duodenum.

Despite aggressive antifungal therapy with amphotericin B and fluconazole, the patient's clinical situation deteriorated and he succumbed to multisystem organ failure one and half months later.

## 3. Discussion

Coccidioidomycosis (also known as valley fever) results from inhaling the spores (arthroconidia) of* Coccidioides* species (*Coccidioides immitis* or* Coccidioides posadasii*) [7]. In the United States, the areas with the highest incidence are primarily in the Sonoran desert in Arizona (Phoenix and Tucson metropolitan areas) and the San Joaquin “Central” Valley in California. Other endemic areas include parts of New Mexico, western Texas, Nevada, and Utah [8]. Our patient had lived previously in San Joaquin Valley.

Most infections are asymptomatic. Symptomatic persons will generally have disease ranging from a self-limited influenza-like illness to primary pulmonary coccidioidomycosis, characterized by pneumonia with changes on chest radiography [9]. Extrapulmonary dissemination is rare and is associated with significant morbidity and mortality. Risk factors for extrapulmonary dissemination include a preponderance of males (nearly 2 : 1), African Americans and Filipinos, those who are immunocompromised (HIV/AIDS and malignancy), and women in the third trimester of pregnancy [4, 10, 11]. Our case is unique in several aspects. First, the patient is immunocompetent. The obvious risk factors for dissemination were his gender and race (African American). Secondly, the patient presented with abdominal distention and pain, joint pain, weight loss, and a rapidly progressing course. For unknown reasons, gastrointestinal coccidioidomycosis is extremely rare [4–6]. Our case shows severe duodenum involvement.

Although coccidioidomycosis is a great imitator, the main difficulty in diagnosis is failure to consider coccidioidomycosis. The diagnosis of coccidioidomycosis can usually be made readily in the following 3 ways [12, 13]: (1) identification of coccidioidal spherules in a cytology or biopsy specimen, (2) culture from any body fluid that is positive for* Coccidioides* spp., or (3) positive serologic tests for antibodies to coccidioidal antigens. The histological presentation of coccidioidomycosis is thick-walled spherules ranging in size from 20 mm to 100 mm in diameter. The spherules are often filled with 2 to 4 mm of endospores. The presence of a mature spherule with endospores in tissue, sputum, bronchoalveolar lavage fluid, or other body fluids or a positive culture from any location in the body is pathognomonic of coccidioidal infection. The endospores are released on rupture of the spherules, causing further dissemination. Growth of the isolated* Coccidioides* from clinical specimen is typically slow, often requiring 7 days or more for sporulation; however, growth as early as 3 or 4 days may occur if the concentrate of fungal forms is high. Humoral antibodies can be used for the diagnosis and prognosis of coccidioidomycosis. Compared with the immunocompetent group, immunosuppressed persons had lower rates of seropositivity [14]. Sequential complement fixation (CF) studies for IgG class of antibody are useful for the prognosis of coccidioidomycosis. Titers exceeding 1 : 16 usually reflect disseminated disease. In general, higher titers are correlated with disease severity. A negative CF test does not, however, rule out the diagnosis. In this case, the coccidioidal complement fixation titer was 1 : 256 in all three tests.

In conclusion, although gastrointestinal coccidioidomycosis is extremely rare, it does exist and a higher index of suspicion should be maintained in patients from an endemic area presenting as abdominal distention and pain.
